# Impact of Bronchiectasis on Long-Term Mortality in Patients with Rheumatoid Arthritis: A Real-World Retrospective Cohort Study

**DOI:** 10.3390/jcm15218703

**Published:** 2026-11-07

**Authors:** Shunsuke Mori, Fumikazu Sakai, Mizue Hasegawa, Kazuyoshi Nakamura

**Affiliations:** 1 Department of Rheumatology, Clinical Research Center for Rheumatic Diseases, National Hospital Organization (NHO) Kumamoto Saishun Medical Center, Kohshi 861-1196, Kumamoto, Japan; 2 Department of Radiology, Kanagawa Cardiovascular and Respiratory Center, Yokohama 236-0051, Kanagawa, Japan; fmksakai@yahoo.co.jp; 3 Department of Respiratory Medicine, Tokyo Women’s Medical University Yachiyo Medical Center, Yachiyo 276-8524, Chiba, Japan; hasemizue@yahoo.co.jp; 4 Department of Respiratory Medicine, NHO Kumamoto Saishun Medical Center, Kohshi 861-1196, Kumamoto, Japan; nakamura.kazuyoshi.cy@mail.hosp.go.jp

**Keywords:** bronchiectasis, rheumatoid arthritis, interstitial lung disease, mortality risk, predictive factors

## Abstract

**Objectives**: This study aimed to examine the impact of bronchiectasis on long-term mortality in patients with rheumatoid arthritis (RA). **Methods**: We used real-world cohort data from patients who were diagnosed with RA at our institution between April 2001 and June 2023. Pulmonary conditions were evaluated using high-resolution computed tomography imaging taken at RA diagnosis and over the disease course. Traction bronchiectasis was not included in the diagnosis of bronchiectasis. **Results**: Among 766 patients with RA, 2.7% were diagnosed with bronchiectasis plus interstitial lung disease (ILD), 5.6% with bronchiectasis alone, and 7.3% with ILD alone. During follow-up with a mean of 9.7 years, the crude incidence rate of death (95% confidence interval [CI]) per 100 person-years was 11.1 (6.7–18.4) in RA patients with bronchiectasis plus ILD; 7.0 (4.6–10.5) in those with bronchiectasis alone; 6.8 (4.6–10.1) in those with ILD alone; and 1.2 (1.0–1.5) in those without either complication. The standardized mortality ratio (95% CI) compared with the general population was 2.57 (1.76–3.39) for RA patients with bronchiectasis; 2.17 (1.50–2.85) for those with ILD; and 0.93 (0.73–1.14) for those without either complication. In multivariable Cox regression analysis using RA diagnosis as the index date and treating pulmonary complications as time-varying exposure variables, the presence of bronchiectasis alone or ILD alone, advanced age, and low body mass index were identified as strong predictive factors for death. The adjusted hazard ratio (95% CI) was 2.37 (1.48–3.82) for bronchiectasis alone and 3.16 (1.92–5.22) for ILD alone. When limited to bronchiectasis patients with and without ILD, the complication of ILD did not influence the mortality risk. **Conclusions**: Bronchiectasis was associated with excess mortality in RA patients, regardless of the complication of ILD. Bronchiectasis and ILD had similar levels of increased mortality risk in RA patients.

## 1. Introduction

Bronchiectasis is a chronic lung disease that is defined radiologically by the presence of permanent bronchial dilatation and clinically by cough, sputum production, and recurrent respiratory infections [1,2]. A wide variety of underlying conditions are known to cause or be associated with bronchiectasis, including post-infectious diseases, chronic obstructive pulmonary disease (COPD), and connective tissue diseases such as rheumatoid arthritis (RA), immune deficiency, asthma, and allergic bronchopulmonary aspergillosis [3,4,5,6]. Multiple etiologies and comorbidities remain major challenges in clinical practice [7,8,9,10].

RA is a chronic autoimmune inflammatory disease that typically affects multiple synovial joints, leading to inflammation, damage, and disability. RA-associated systemic inflammation and immune reactivity also cause extra-articular damage to various tissues and organs, which can affect the natural course of RA [11,12,13]. With advancements in RA management during recent decades, RA-related mortality is decreasing over time. However, a persistent mortality gap remains between RA patients and the general population or non-RA controls [14,15,16,17,18]. Cardiovascular disease, malignancy, infectious disease, and pulmonary disease are significantly linked to excess RA-related mortality [16,18,19,20,21,22,23].

One of the most common extra-articular manifestations of RA is pulmonary involvement, where almost every compartment of the pulmonary system can be affected [24,25,26]. The reported prevalence of pulmonary manifestations varies significantly across studies, but interstitial lung disease (ILD) and bronchiectasis are the most common phenotypes of RA-associated lung disease [27,28]. Recent population-based cohort studies showed increased mortality in RA patients with ILD compared with those without ILD [29,30,31,32,33,34]. In a 10-year follow-up study, after adjusting for potential confounders, we found that the mortality risk was 3 times higher in RA patients with ILD than in those without ILD [35]. Regarding bronchiectasis, a recent nationwide cohort study in Korea showed that RA patients had twice the risk of developing bronchiectasis compared with matched control individuals [36]. However, there is limited information regarding the association of bronchiectasis with long-term mortality in RA patients.

In the present study, we examined the impact of bronchiectasis on long-term mortality in RA patients. Based on pulmonary high-resolution computed tomography (HRCT) imaging taken at RA diagnosis and during the disease course, we classified RA patients as having bronchiectasis plus ILD, bronchiectasis alone, ILD alone, or not having either complication. Traction bronchiectasis was not included in the diagnosis of bronchiectasis. Long-term mortality estimates were determined and compared among the four patient groups. To examine the contribution of bronchiectasis to excess RA-related mortality, we calculated standardized mortality ratios (SMRs) in RA patients with bronchiectasis compared with the general population. Using Cox proportional hazards regression analysis, we identified predictive factors for death in overall RA patients as well as in bronchiectasis patients with and without ILD.

## 2. Materials and Methods

### 2.1. Patients

**Figure 1:**
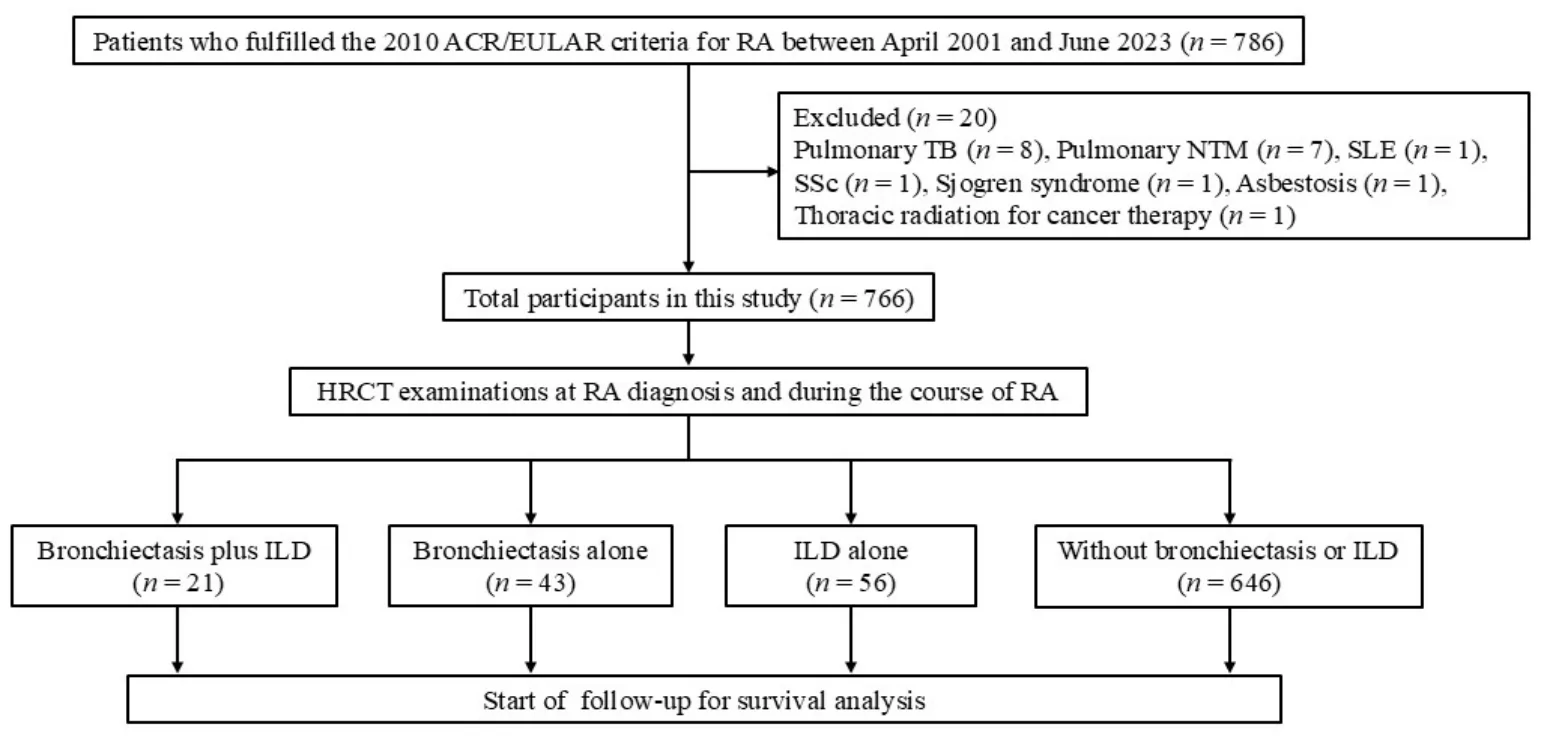
Participant enrollment flow diagram. The diagnosis of bronchiectasis was defined by the presence of bronchiectasis separately from areas of interstitial abnormalities. Patients with ILD and traction bronchiectasis on HRCT scans were not classified as having bronchiectasis plus ILD, but rather classified as having ILD alone. Abbreviations: ACR, American College of Rheumatology; EULAR, European Alliance of Associations for Rheumatology; HRCT, high-resolution computed tomography; ILD, interstitial lung disease; NTM, non-tuberculous mycobacteria; RA, rheumatoid arthritis; SLE, systemic lupus erythematosus; SSc, systemic sclerosis; TB, tuberculosis.

To examine clinical features and long-term outcomes in RA patients with bronchiectasis, we used a real-world database of patients who received a diagnosis of RA at the rheumatology division of National Hospital Organization (NHO) Kumamoto Saishun Medical Center between April 2001 and June 2023. All participants were required to be ≥18 years of age and to fulfill the 2010 American College of Rheumatology (ACR)/European Alliance of Associations for Rheumatology (EULAR) criteria for diagnosis of RA [37]. The patient enrollment flow diagram is shown in Figure 1. Patients were excluded from this study if they had any of the following histories, because these conditions could cause bronchiectasis or ILD that was not associated with RA: (1) other collagen vascular diseases/autoimmune diseases; (2) pulmonary nontuberculous mycobacterial (NTM) disease; (3) pulmonary tuberculosis; (4) exposure to dust such as asbestos or silica; or (5) thoracic radiation for cancer therapy. Based on smoking history, clinical symptoms/signs, HRCT findings, pulmonary function test (PFT) data, bronchodilator testing, allergy testing, or combinations thereof, we confirmed that no participants had asthma, COPD, or allergic bronchopulmonary aspergillosis at enrollment.

### 2.2. Study Design

The diagnosis of bronchiectasis and ILD was made based on chest HRCT images taken at RA diagnosis and over the course of RA. Pulmonary HRCT examinations were performed every 3 years during follow-up or at any time if patients had pulmonary symptoms or signs that were indicative of the development of pulmonary diseases. In addition, chest radiography was performed at least every year, and if abnormal findings were observed, HRCT was performed immediately. Each participant was classified as having bronchiectasis plus ILD, bronchiectasis alone, or ILD alone. Bronchiectasis can occur in areas demonstrating interstitial lung abnormalities such as pulmonary fibrosis or distorted lung parenchymal architecture (termed “traction bronchiectasis”). In this study, traction bronchiectasis was not included in the diagnosis of bronchiectasis. The diagnosis of bronchiectasis in this study was defined by the presence of bronchiectasis that occurred separately from areas of interstitial abnormalities on HRCT scans. Accordingly, patients with ILD and traction bronchiectasis on HRCT scans were not classified as having bronchiectasis plus ILD, but rather were classified as having ILD alone.

We reviewed patient medical records to collect clinical data at the time of RA diagnosis and HRCT-based diagnosis of pulmonary complications. The following data were collected: demographic characteristics, RA-related data (disease duration, disease activity, Steinbrocker radiological stage, and positivity for anti-cyclic citrullinated peptide antibodies [anti-CCP] and rheumatoid factor [RF]), smoking history, body mass index (BMI), and comorbidities (diabetes mellitus, malignancy history, and cardiovascular disease history). Considering the availability of disease-modifying antirheumatic drugs (DMARDs), the entry year was also recorded. The number and duration of DMARDs were extracted from patients’ medical records.

Follow-up started on the day of HRCT-based diagnosis of bronchiectasis plus ILD, bronchiectasis alone, or ILD alone. For patients without bronchiectasis or ILD, follow-up began with the diagnosis of RA. For all participants, follow-up ended with death, loss to follow-up, or the last visit before 31 December 2023, whichever was first. Participants who were transferred to other hospitals or missed two or more scheduled visits were classified as lost to follow-up. The cause of death was determined according to each treating physician’s judgment.

### 2.3. Pulmonary Function Tests

For RA patients with bronchiectasis, we collected PFT data when patients were diagnosed with bronchiectasis plus ILD or bronchiectasis alone. Forced vital capacity (FVC), forced expiratory volume in the first second (FEV1), maximal mid-expiratory flow (MMF), and diffusing capacity of carbon monoxide (DLco) were measured using a rolling-seal-type spirometer. Results were expressed as a ratio of measured to normal predicted values (% predicted). An abnormal PFT was defined as a % predicted value <80% for each PFT. The FEV1/FVC ratio was also calculated; an abnormal FEV1/FVC ratio was defined as <70%. Detailed methods for evaluation of PFT data were previously described [38]. Exacerbation of ILD was defined in our previous study [39].

### 2.4. HRCT-Based Diagnosis of Bronchiectasis and ILD

Pulmonary HRCT images taken at RA diagnosis and during the course of RA were collected from our database and viewed in random order and independently by two observers (one board-certified radiologist [F. Sakai] and one board-certified pulmonologist [M. Hasegawa]). The readers were blinded to the participants’ clinical status, PFT results, and treatment for RA. We identified the following HRCT abnormalities: bronchiectasis/bronchiolectasis, centrilobular nodules, mosaic attenuation, bronchial wall thickening, cysts, ground-glass opacity, intralobular reticular opacity, airspace consolidation, honeycombing, traction bronchiectasis, architectural distortion, and emphysema. The inter-observer agreement between the two readers was determined using the k coefficient.

Radiological diagnosis of bronchiectasis was made if participants had at least one of the following findings on pulmonary HRCT scans: (1) an inner airway-artery diameter ratio of ≥1.0; (2) an outer airway-artery diameter ratio of ≥1.0; (3) a lack of tapering of the airways; or (4) visibility of airways in the periphery [1]. Traction bronchiectasis was not used for the diagnosis of bronchiectasis.

Radiological diagnosis of ILD was made according to the American Thoracic Society (ATS)/European Respiratory Society (ERS)/Japanese Respiratory Society (JRS)/Latin American Thoracic Society (ALAT) official clinical practice guidelines for the diagnosis of idiopathic pulmonary fibrosis [40,41]. Detailed criteria for the diagnosis of ILD are described elsewhere [35].

### 2.5. Clinically Significant Bronchiectasis

The clinical diagnosis of bronchiectasis was defined as the presence of at least two of the following: (1) a cough most days of the week; (2) sputum production most days of the week; or (3) a history of exacerbations. Clinically significant bronchiectasis was defined as the presence of both radiological and clinical evidence of bronchiectasis [1].

### 2.6. Severity of Bronchiectasis

The bronchiectasis severity index (BSI) was determined for each participant who had a diagnosis of HRCT-proven bronchiectasis [42]. The severity markers included the following nine variables: increasing age, BMI < 18.5, FEV1, severe exacerbations in the previous year, exacerbation frequency in the previous year (annual frequency), Medical Research Council (MRC) dyspnea score, colonization with *Pseudomonas aeruginosa*, colonization with other microorganisms, and radiological severity defined as the presence of ≥3 lobes affected or cystic changes. An exacerbation was defined as a worsening of symptoms that exceeded day-to-day variability and required a change in management. Core symptoms of exacerbation include a change in cough, sputum volume and/or consistency, sputum purulence, dyspnea and/or exercise intolerance, fatigue or malaise, and hemoptysis. A severe exacerbation of bronchiectasis was defined as requiring hospitalization or intravenous antibiotic treatment [2].

### 2.7. Statistical Analysis

Mean and standard deviation (SD) were used as descriptive statistics for continuously distributed data, which included non-normally distributed data [43], and numbers (percentages) were used for categorical data. To compare data among the four disease groups, we performed Fisher’s exact probability test with the post hoc Holm test for categorical variables and one-way analysis of variance (ANOVA) with Tukey’s honestly significant difference (HSD) post hoc test for continuous variables. Comparisons of data between the bronchiectasis groups with and without ILD were performed using Fisher’s exact probability test for categorical variables and using the independent-measures *t*-test for continuous variables. For data that deviated substantially from the normal distribution, we used the Kruskal–Wallis test with Tukey’s HSD post hoc test or the Mann–Whitney U test. No participants had missing data for measurements at the time of RA diagnosis and pulmonary disease diagnosis.

Crude incidence rates of death and 95% confidence intervals (CIs) were calculated by dividing the number of incidence cases (death cases) by the number of corresponding follow-up person-years (PYs).

SMRs and 95% CIs were calculated as the ratio of the observed number of deaths in our cohort divided by the expected number of deaths in the reference population from 2001 to 2023. For the reference population, we used mortality data in the Japanese general population reported by the Ministry of Health, Labour and Welfare in Japan (http://www.stat.go.jp/english/index.html). Expected numbers of deaths in the reference population were calculated based on age- and sex-specific population mortality rates in each calendar year for patients at risk during follow-up.

The probability of overall survival was estimated using the Kaplan–Meier approach. The log-rank test with a post hoc Holm test was used to test the equality of Kaplan–Meier survival estimates over time among the four disease groups. Cox proportional hazards regression analysis was performed to evaluate the effect of each patient characteristic on death over time and to calculate adjusted hazard ratios (HRs) with 95% CIs. As predictor variables, we used a set of patient characteristics that were considered clinically relevant and important based on previous knowledge, which included demographic data, RA-related data, lung disease diagnosed by HRCT scans, smoking history, BMI, and entry year. For RA patients with bronchiectasis plus ILD, bronchiectasis alone, or ILD alone, the index date was defined as the time of diagnosis of each pulmonary disease. For RA patients without either pulmonary complication, the index date was defined as the time of diagnosis of RA. In the sensitivity analysis, we used the common index date (the time of RA diagnosis) as the start of follow-up for overall RA patients and treated each pulmonary disease as a time-varying exposure variable. Predictor variables with *p*-values < 0.10 in univariable models were introduced into a multivariable Cox regression analysis. A forced entry procedure was used in the multivariable model. For regression analyses limited to bronchiectasis patients, timing of bronchiectasis onset, BSI, clinically significant bronchiectasis, MRC dyspnea score, HRCT abnormal findings, and PFT data were additionally used as predictor variables. Because RA and ILD were diagnosed before or simultaneously with the diagnosis of bronchiectasis, the index date was defined as the time of diagnosis of bronchiectasis. We confirmed the proportional hazards assumption using log-minus-log plots for exposure variables over time.

Two-sided *p*-values < 0.05 were considered to indicate statistical significance. All calculations were performed using PASW Statistics version 27 (SPSS Japan Inc., Tokyo, Japan) and Easy R version 1.70 (Saitama Medical Center, Jichi Medical University, Saitama, Japan) [44].

## 3. Results

### 3.1. Characteristics of RA Patients, Grouped According to HRCT-Based Diagnosis of Lung Disease

A total of 766 patients with RA were enrolled in this study (Figure 1). Based on HRCT scans taken at diagnosis and during the course of RA, 64 patients (8.4%) received a diagnosis of bronchiectasis with and without ILD (21 patients [2.7%] with bronchiectasis plus ILD and 43 patients [5.6%] with bronchiectasis alone). Additionally, 56 patients (7.3%) had a diagnosis of ILD alone. The remaining 646 patients were diagnosed without bronchiectasis or ILD. Patient characteristics are shown in Table 1. Age was significantly higher in RA patients with bronchiectasis and/or ILD compared with those without either complication. Male patients were predominant in the groups with ILD compared with the groups without ILD. Smoking history was also significantly associated with the complication of ILD. Anti-CCP and RF were present in patients with bronchiectasis and/or ILD at higher rates than in those without either complication. BMI < 18.5 was also more frequently observed in patients with bronchiectasis and/or ILD compared with those without either complication. Advanced radiological stages (Steinbrocker stages III/IV) were more often observed in the bronchiectasis alone group compared with the other three groups. Disease duration was longer in bronchiectasis patients than in patients without bronchiectasis. An entry year from 2001 to 2011 was more likely to be observed in the bronchiectasis alone group compared with the other three groups.

### 3.2. Characteristics of HRCT-Proven Bronchiectasis in RA Patients: Comparisons Between Bronchiectasis Plus ILD and Bronchiectasis Alone

Characteristics of patients with RA and bronchiectasis are shown in Table 2. Among these patients, 29.7% received a simultaneous diagnosis of RA and bronchiectasis. The mean BSI was 5.0 and only one patient had severe BSI at the time of bronchiectasis diagnosis. Detailed data on each component of BSI are shown in Table S1. Age and BMI were major determinants of BSI. Symptomatic bronchiectasis was observed in 26.6% of bronchiectasis patients, and no patients experienced exacerbation of bronchiectasis in the previous year. In almost all cases, the MRC dyspnea score was ≤3. There was no significant difference in the above-mentioned characteristics between bronchiectasis patients with ILD and those without ILD.

Regarding HRCT findings, approximately 90% of patients with and without ILD had ≥3 lobes involved in bronchiectasis (90.5% vs. 88.4%, Table S1). As shown in Table 2, centrilobular nodules and bronchial wall thickness, which suggested the presence of bronchiolitis, were detected in most patients with bronchiectasis (75% and 95.3%, respectively). Centrilobular nodules were present more frequently in patients with bronchiectasis alone compared with those with bronchiectasis plus ILD (88.3% vs. 47.6%). Mosaic attenuation, which suggested the presence of constrictive bronchiolitis, was seen in 12.5% of bronchiectasis patients. Mosaic attenuation was more likely to exist in patients with bronchiectasis plus ILD compared with those with bronchiectasis alone (28.6% vs. 4.7%), which may be explained by an association between mosaic attenuation and smoking-related ILD in RA.

Regarding PFT, the mean values of % predicted for FVC, FEV1, and FEV1/FVC ratio were within the normal range in bronchiectasis patients with ILD and those without ILD (Table 2). In contrast, the mean % predicted for MMF, a sensitive marker of obstruction of smaller airways, was 60.1%, and it was significantly lower in patients with bronchiectasis alone than in those with bronchiectasis plus ILD (51.4% vs. 78.1%). The mean % predicted for DL_CO_, an index of restrictive interstitial disease, was significantly lower in patients with bronchiectasis plus ILD compared with bronchiectasis patients without ILD (78.9% vs. 95.2%).

### 3.3. Mortality in RA Patients

Follow-up began when the diagnosis of each pulmonary complication or RA diagnosis was made (mean of 9.7 years). As shown in Table 3, death events occurred in 144 patients (18.8%); the mortality of each group was 71.4% for bronchiectasis plus ILD, 53.5% for bronchiectasis alone, 44.6% for ILD alone, and 12.5% for the group without bronchiectasis or ILD. The crude incidence rate of death was significantly higher in patients with bronchiectasis and/or ILD compared with those without bronchiectasis or ILD (11.1 per 100 PYs [95% CI, 6.7–18.4] for bronchiectasis plus ILD, 7.0 per 100 PYs [95% CI, 4.6–10.5] for bronchiectasis alone, and 6.8 per 100 PYs [95% CI, 4.6–10.1] for ILD alone vs. 1.2 per 100 PYs [95% CI, 1.0–1.5] for patients without bronchiectasis or ILD). When bronchiectasis patients were grouped according to the presence of clinically significant bronchiectasis, there was no significant difference in mortality between symptomatic and asymptomatic bronchiectasis. The crude incidence rate of death was 11.7 per 100 PYs (95% CI, 6.9–19.8) for symptomatic patients and 7.2 per 100 PYs (95% CI, 4.9–10.7) for asymptomatic patients.

**Figure 2:**
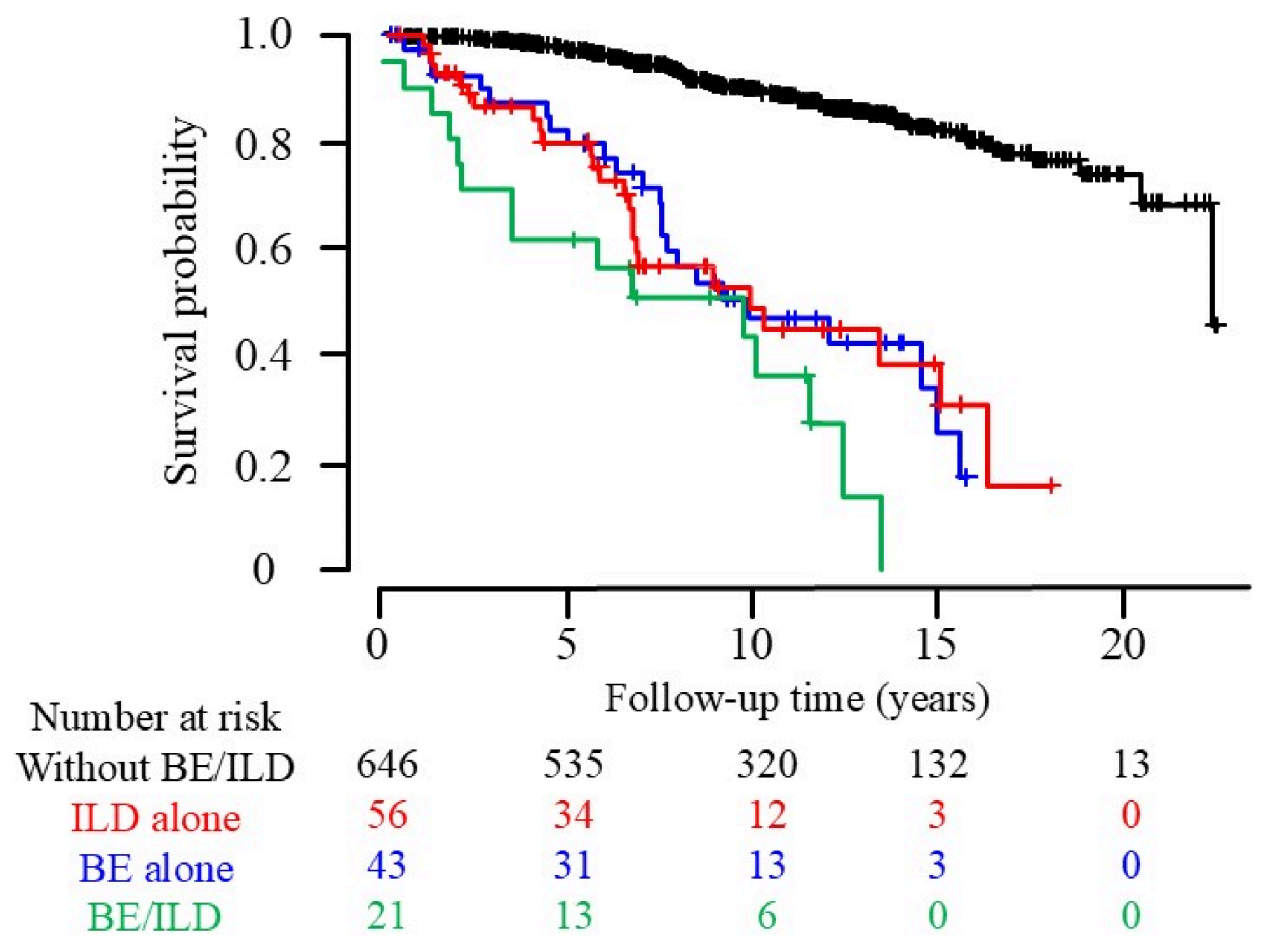
Kaplan–Meier survival curves. Survival probabilities are shown grouped according to HRCT-based diagnosis of lung disease. Numbers below the figure represent the numbers of patients at risk. Abbreviations: BE, bronchiectasis; HRCT, high-resolution computed tomography; ILD, interstitial lung disease.

As shown in Figure 2, significant differences in Kaplan–Meier survival estimates over time were observed between the bronchiectasis plus ILD, bronchiectasis alone, and ILD alone groups and the group without either pulmonary complication (*p* < 0.001 with log-rank test and Holm post hoc test).

### 3.4. SMRs for RA Patients Compared with the General Population of Japan

The SMRs (95% CIs) in our RA cohort compared with the general population of Japan were 1.29 (1.08–1.50) for overall RA patients, 2.57 (1.76–3.39) for RA patients with bronchiectasis, 2.17 (1.50–2.85) for RA patients with ILD, and 0.93 (0.73–1.14) for RA patients without bronchiectasis or ILD. Detailed data are shown in Table 4.

### 3.5. Cause of Death in RA Patients

Among RA patients without bronchiectasis or ILD, malignancy was the most common cause of death, followed by cardiovascular events, serious infection, and old age (34.6%, 22.2%, 11.1%, and 11.1% of death cases, respectively; Table 5). While no patients in the group without bronchiectasis or ILD died from respiratory failure, considerable proportions of patients with bronchiectasis and/or ILD died of respiratory failure due to acute exacerbation of ILD (26.7% of death cases in the bronchiectasis plus ILD group and 32.0% in the ILD alone group) or due to severe exacerbation of bronchiectasis (13% of death cases in the bronchiectasis alone group). Pulmonary NTM disease occurred in three patients with bronchiectasis plus ILD and three patients with bronchiectasis alone during follow-up. The three patients with bronchiectasis alone died due to severe exacerbation of bronchiectasis. The cause of ILD exacerbation was not identified in any case. Death due to lung cancer was more frequently observed in RA patients with ILD (40% in the bronchiectasis plus ILD group and 16% in the ILD alone group) compared with those without ILD (0% in the bronchiectasis alone group and 3.7% in the group without bronchiectasis or ILD). Based on chest radiological images obtained immediately before death, we confirmed that RA patients in the group without bronchiectasis or ILD did not develop either complication by the time of death.

### 3.6. Predictive Factors for Death in RA Patients Overall

Of the 766 RA patients overall, there were 144 deaths. Using Cox proportional hazards regression analysis, we examined the effect of clinically important characteristics on mortality in overall RA patients (Table 6). All predictor variables with *p*-values < 0.10 in the univariable analyses (10 variables) were introduced into the multivariable analysis using a forced entry procedure. Through multivariate regression modeling, age (adjusted HR 1.10 per additional year [95% CI, 1.08–1.12], *p* < 0.001); presence of HRCT-proven lung disease (bronchiectasis plus ILD, adjusted HR 4.12 [95% CI 2.13–7.95]; bronchiectasis alone, 4.43 [2.56–7.66]; ILD alone, 3.60 [2.19–5.93, *p* < 0.001); and BMI < 18.5 (adjusted HR 2.84 [95% CI, 1.86–4.34], *p* < 0.001) were identified as independent predictive factors for death.

### 3.7. Sensitivity Analysis

More than 80% of RA patients with ILD received a simultaneous diagnosis of RA and ILD. Regarding bronchiectasis, 29.7% of patients received a simultaneous diagnosis of RA and bronchiectasis, and 26.6% of patients were diagnosed with bronchiectasis within 3 years of RA diagnosis. In the remaining patients, the interval between RA and bronchiectasis diagnoses was more than 3 years. Considering these findings, we conducted additional Kaplan–Meier and Cox regression analyses of the overall RA patient population, using a common index date (RA diagnosis) as the start of follow-up and treating the diagnosis of each pulmonary disease as a time-varying exposure variable.

**Figure 3:**
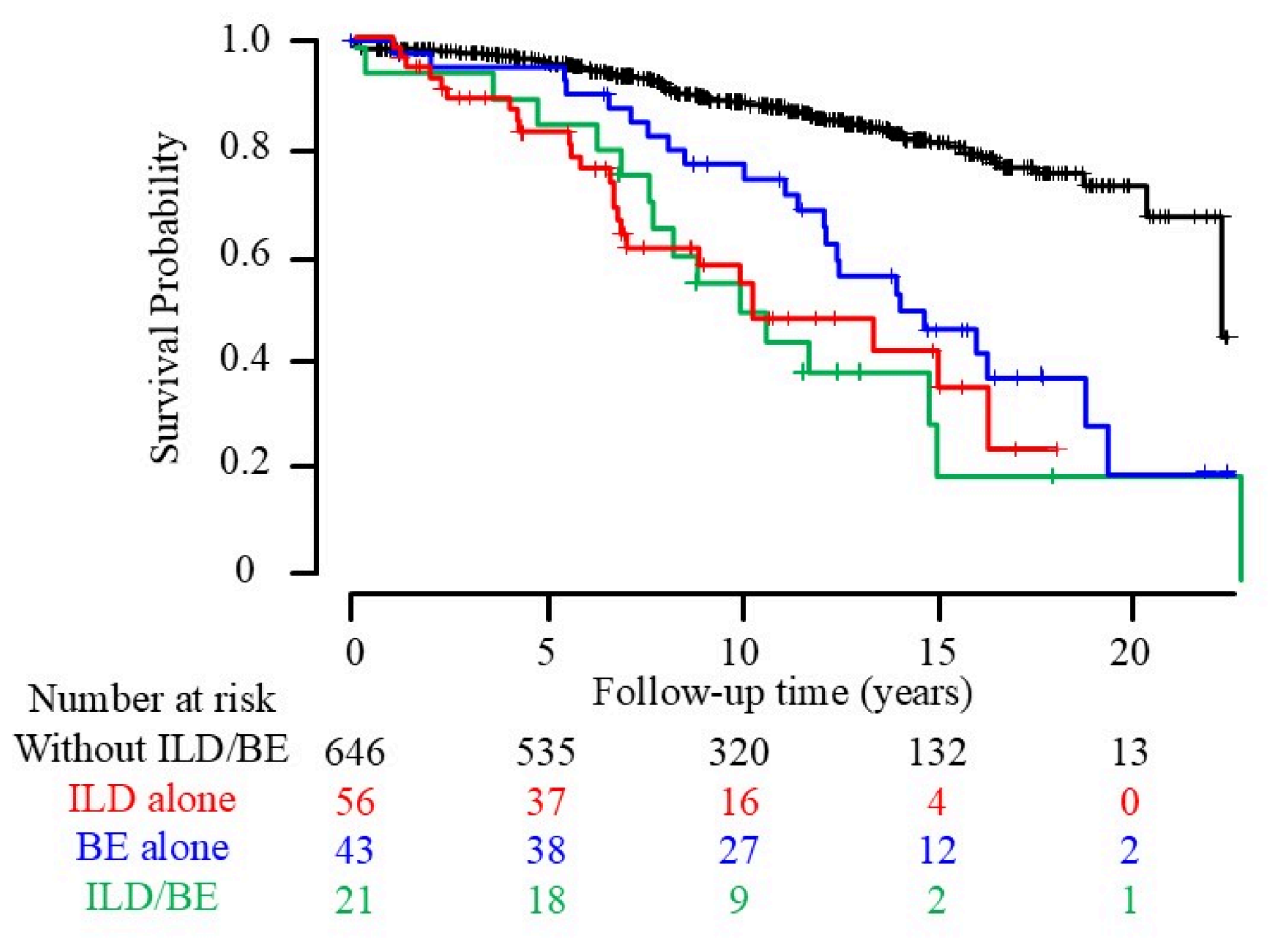
Kaplan–Meier survival curves (sensitivity analysis). Survival probabilities are shown grouped according to HRCT-based diagnosis of lung disease. The common index date (RA diagnosis) was used as the start of follow-up. Numbers below the figure represent the numbers of patients at risk. Abbreviations: BE, bronchiectasis; HRCT, high-resolution computed tomography; ILD, interstitial lung disease.

As shown in Figure 3, there were significant differences in Kaplan–Meier survival estimates over time between the bronchiectasis plus ILD, bronchiectasis alone, and ILD alone groups versus the group without either pulmonary complication (*p* < 0.001 with log-rank test and Holm post hoc test).

As shown in Table 7, all predictor variables with *p*-values < 0.10 in the univariable analyses (7 variables) were introduced into the multivariable analysis using a forced entry procedure. The results obtained were similar to the data shown in Table 6. The presence of bronchiectasis alone or ILD alone, advanced age, and BMI < 18.5 were identified as strong predictive factors for death. Adjusted hazard ratios (95% CI) were 2.37 (1.48–3.82) for bronchiectasis alone (*p* < 0.001); 3.16 (1.92–5.22) for ILD alone (*p* < 0.001); 1.10 (1.08–1.12) per additional year for advanced age (*p* < 0.001); and 2.68 (1.75–4.11) for BMI < 18.5 (*p* < 0.001). The presence of bronchiectasis plus ILD did not reach statistical significance (adjusted HR, 1.62; 95% CI, 0.89–2.94; *p* = 0.12), which may be explained in part by the small number of patients in this pulmonary disease group.

### 3.8. Predictive Factors for Death in RA Patients with Bronchiectasis

Among 64 patients with bronchiectasis, there were 38 death events. Results of Cox regression analyses for RA patients with bronchiectasis are shown in Table 8. For the multivariable analysis, we used BSI as one predictor variable with *p*-value < 0.10 in the univariable analysis. Age, BMI, and MRC dyspnea score were also identified as predictive variables with *p*-values < 0.10 in the univariable analysis. However, we excluded these patient characteristics from the multivariable analysis because they are components of BSI. Considering the small number of death events, we used BSI as a single predictor variable instead of introducing age, BMI, and MRC dyspnea score into the multivariable analysis. As a result, three predictor variables were introduced into the multivariable regression analysis using a forced entry procedure. Through multivariable regression modeling, we found that BSI was the only significant predictive factor for death in RA patients with bronchiectasis (adjusted HR 1.37 per additional unit [95% CI, 1.20–1.58], *p* < 0.001). However, we could not interpret the association between BSI and mortality independently of the BSI components, particularly age and BMI. Considering that age and BMI were major determinants of BSI in our patients (Table S1), the effect of BSI on mortality risk was probably attributable to advanced age and low BMI. After adjustment for potential confounders, ILD was not identified as a factor associated with death in RA patients with bronchiectasis.

### 3.9. DMARD Use During Follow-Up

In our cohort, all patients started DMARD therapy for RA under a tight-control strategy. As shown in Table S2, 85.9% of RA patients received methotrexate (MTX) as monotherapy or in combination with other DMARDs during follow-up. Compared with patients without bronchiectasis or ILD, the rates of MTX use were lower in patients with bronchiectasis and/or ILD. Instead of MTX treatment, these patients were more often prescribed other conventional synthetic DMARDs (csDMARDs). The rates of tumor necrosis factor (TNF) inhibitor users and Janus kinase (JAK) inhibitor users were similar among the four patient groups, but the use of interleukin-6 (IL-6) inhibitors and abatacept varied. Exposure duration to all DMARDs except MTX was similar among the four patient groups.

## 4. Discussion

In our RA cohort, HRCT-proven bronchiectasis was found in 8.4% of patients overall. During follow-up with a mean of 9.7 years, the crude incidence rate of death was 6 to 9 times higher in RA patients with bronchiectasis and/or ILD than in those without either pulmonary complication. Compared with the general population, SMRs indicated that the complication of bronchiectasis or ILD was associated with RA-related excess mortality. In the multivariable Cox regression analysis for overall RA patients, advanced age, low BMI, and the presence of bronchiectasis or ILD were identified as strong predictive factors for death; adjusted hazard ratios were 2.37 for bronchiectasis alone and 3.16 for ILD alone compared with patients without either pulmonary complication. When limited to RA patients with bronchiectasis, the complication of ILD did not influence the mortality risk in this patient group.

Using bronchiectasis databases across six medical centers in European countries (including 147 patients with an overlap syndrome of RA and bronchiectasis [BROS] without ILD, 877 with other-cause bronchiectasis, and 692 with idiopathic bronchiectasis), De Soyza et al. estimated the mortality rates over a mean period of 48 months to be 18% for BROS and 9.3% for idiopathic bronchiectasis. The adjusted odds ratio of RA for death was 2.03 (95% CI, 1.19–3.44), which suggested that RA was independently associated with poor outcomes in bronchiectasis patients without ILD [45]. In the present study, the 5-year mortality rate for RA patients with bronchiectasis alone was estimated to be 19.5%, which was similar to the 48-month mortality estimate for patients with BROS. Through the multivariable Cox regression analysis for RA patients with bronchiectasis with and without ILD, the complication of ILD had little impact on the mortality risk in this patient group, which indicated that regardless of the presence or absence of ILD, bronchiectasis can increase the mortality risk in RA patients.

Using a Korean National Health Insurance Service Database including 3355 RA patients with bronchiectasis (BROS) and 16,240 matched controls with bronchiectasis only between 2010 and 2017, Choi et al. showed that BROS patients had a 2.09-fold higher mortality risk compared with patients with bronchiectasis only, even after adjusting for potential confounders (95% CI, 1.88–2.33). The increased risk of death was more evident in seropositive RA patients; the HRs of death for seropositive and seronegative RA patients were 2.34 (95% CI, 2.09–2.62) and 1.29 (95% CI, 1.01–1.65), respectively. The authors suggested that RA-related inflammation may affect disease progression and mortality, although that study included no information on disease activity or severity [46]. In the present study, anti-CCP positivity and RF positivity were both identified as predictive factors for death in the univariable Cox regression analyses for RA patients with bronchiectasis. After adjusting for potential confounders, however, seropositivity of these factors did not remain a significant predictive factor for death.

In a multicenter cohort study including 343 RA patients with bronchiectasis (BROS patients) at 7 medical centers and hospitals in Taiwan between 2006 and 2017, Lin et al. showed that the 3-year mortality rate was 25.7%, although it was not clear whether their BROS patients had concurrent ILD in addition to bronchiectasis. The BROS patients who had been diagnosed with RA before diagnosis of bronchiectasis showed a significantly higher mortality rate at 3 years compared with those who had been diagnosed with bronchiectasis before RA (30.4% vs. 15.9%) [47]. In the present study, however, diagnosis chronology (onset of bronchiectasis and RA) was not identified as a predictive factor for death in RA patients with bronchiectasis, even in the univariable Cox regression analysis. Lin et al. suggested that poor outcomes in patients with RA diagnosed before the bronchiectasis diagnosis might reflect the higher severity of RA in this patient group compared with those in whom RA was diagnosed after bronchiectasis diagnosis. In our RA cohort, however, a difference in RA severity according to the diagnosis timing of bronchiectasis was not observed.

In a retrospective cohort study using data from 70 RA patients with bronchiectasis, 151 with ILD, and 980 without either pulmonary disease in the Mass General Brigham Biobank, Zhang et al. showed that bronchiectasis patients and ILD patients had 1.36-fold (95% CI 0.79–2.35) and 2.27-fold (95% CI 1.61–3.19) increased mortality risks, respectively, compared with patients without bronchiectasis or ILD. Mortality risk due to respiratory disease was significantly higher in bronchiectasis patients and ILD patients than in patients without either pulmonary disease [48]. In the present study, we found that the presence of bronchiectasis or ILD was associated with increased mortality risk in overall RA patients: the HRs (95% CI) of bronchiectasis alone and ILD alone for death were 2.37 (1.48–3.82) and 3.16 (1.92–5.22), respectively, compared with patients without bronchiectasis or ILD. Bronchiectasis and ILD showed similar levels of increased mortality risk. In addition, we found that death from respiratory failure occurred only in RA patients with bronchiectasis and/or ILD. Zhang et al. showed that bronchiectasis patients and ILD patients had a 3-fold higher risk of cancer-related mortality and a 4-fold higher risk of lung infection-related mortality, respectively. In the present study, such differences were not found among the four patient groups.

Previous cohort studies have reported that cardiovascular disease, malignancy, infectious disease, and pulmonary disease contribute to excess mortality in RA patients [16,18,19,20,21,22]. In our RA patients without bronchiectasis or ILD, malignancy was the most common cause of death, followed by cardiovascular events, serious infection, and old age, which are the top four causes of death in the general population of Japan. When compared with the general population, RA patients without bronchiectasis or ILD did not show excess mortality (SMR, 0.93; 95% CI, 0.73–1.14). In contrast, the SMRs were 2.57 (1.76–3.39) for RA patients with bronchiectasis and 2.17 (1.50–2.85) for RA patients with ILD. These data indicate that the excess mortality in RA patients was linked to the complication of bronchiectasis or ILD.

In the present study, all patients who died from bronchiectasis exacerbation developed pulmonary NTM disease during follow-up, although these patients did not receive a diagnosis of NTM infection at enrollment. A recent study showed that NTM infection is associated with the risk of respiratory failure and mortality in bronchiectasis patients [49]. In addition, RA patients are at increased risk for development of pulmonary NTM; the risk of developing NTM disease was 6.24-fold higher among RA patients than among the general population of Taiwan [50]. Considering these findings, RA patients at increased risk of NTM infection should be identified based on clinical features and radiological findings indicative of bronchiectasis and be considered for more frequent follow-up. In the present study, we showed that regardless of the complication of bronchiectasis, lung cancer caused death most frequently in RA patients with ILD. In previous studies, we showed that the presence of ILD is a strong predictive factor for development of lung cancer and lung cancer-related death in RA patients [51,52]. Considering these findings, we should consider close monitoring of RA patients with ILD.

There are several limitations to this study. First, this was a retrospective long-term follow-up study with a mean follow-up of 9.7 years, which may confer certain inherent limitations. Using the electronic medical database in our institution, however, we obtained accurate data on clinical features, PFTs, and HRCT scans at diagnosis of bronchiectasis, ILD, and RA, as well as time from each diagnosis to death, causes of death, and information about DMARD use. Second, this was a single-center cohort study in Japan, which may limit generalization to other populations. Third, it was difficult to include DMARD use in the Cox regression analysis as a predictor variable for death because DMARD use was a time-varying variable. For tight control of RA disease activity, we modified or changed DMARD therapies if patients failed to achieve remission or at least low disease activity within 3 months or if patients suffered from a disease flare. As a result of this strict strategy, most patients received multiple DMARDs for various exposure times from various starting points to various discontinuation points during follow-up. We could not include times between diagnosis and treatment initiation for individual DMARDs or their treatment times as exposure history. Fourth, 18.9% of patients without bronchiectasis or ILD were lost to follow-up, which might have introduced bias into the study. Generally, RA disease activity is well controlled in patients without any extra-articular manifestations. In the present study, most participants lost to follow-up in the group without bronchiectasis or ILD were those who were transferred to other hospitals or clinics because they maintained remission or low disease activity. We confirmed that they did not develop bronchiectasis or ILD at the time of transfer. Considering these points, we assumed that loss to follow-up was unlikely to negate the differences observed between the groups with bronchiectasis and/or ILD and the group without either complication. Lastly, in the present study, baseline RA disease activity was not associated with mortality risk in RA patients. It was difficult to determine the cumulative effects of RA disease activity during follow-up because it fluctuated depending on patients’ responsiveness to DMARD therapies.

## 5. Conclusions

The complications of bronchiectasis and ILD were associated with increased mortality risk in RA patients. Bronchiectasis and ILD had similar levels of mortality risk. For patients with bronchiectasis, the additional complication of ILD had little impact on mortality risk. Compared with the general population, RA patients without bronchiectasis or ILD did not have excess mortality. Respiratory failure was a specific cause of death in RA patients with bronchiectasis and/or ILD. Careful follow-up is expected to result in early and adequate management of exacerbations of bronchiectasis and ILD. Further large-scale studies are warranted to establish multidisciplinary management strategies for RA patients with bronchiectasis, ILD, or a combination of both.

## Figures and Tables

**Table 1:** Characteristics of RA patients, grouped according to HRCT-based diagnosis of lung disease.

	Total	Bronchiectasis Plus ILD ^¶^	Bronchiectasis Alone	ILD Alone ^¶^	Without ILD or Bronchiectasis	
Characteristic	(*n* = 766)	(*n* = 21)	(*n* = 43)	(*n* = 56)	(*n* = 646)	*p* *
Age, years, mean (SD)	60.7 (13.2)	74.6 (8.8)	69.1 (9.9)	69.9 (9.2)	59.0 (13.0)	<0.001
Male, number (%)	201 (26.2)	12 (57.1)	8 (18.6)	31 (55.4)	150 (23.2)	<0.001
Disease duration ^†^, years, mean (SD)	4.1 (7.8)	7.9 (10.5)	10.1 (8.0)	4.4 (7.9)	3.5 (7.5)	<0.001
DAS28-CRP, index, mean (SD)	4.3 (1.3)	4.1 (1.0)	4.3 (1.3)	4.2 (1.1)	4.3 (1.3)	0.080
Steinbrocker stage III/IV, number (%)	259 (33.8)	9 (42.9)	31 (72.1)	21 (37.5)	198 (30.7)	<0.001
Anti-CCP positive, number (%)	668 (87.2)	20 (95.2)	41 (95.3)	54 (96.4)	553 (85.6)	0.023
RF positive, number (%)	669 (87.3)	20 (95.2)	42 (97.7)	55 (98.2)	552 (85.4)	0.004
Smoking ≥ 30 pack-years ^‡^, number (%)	101 (13.2)	8 (38.1)	4 (9.3)	24 (42.9)	65 (10.1)	<0.001
BMI < 18.5, number (%)	80 (10.4)	7 (33.3)	13 (30.2)	9 (16.1)	51 (8.5)	<0.001
Type 2 diabetes, number (%)	94 (12.3)	0	1 (2.3)	9 (16.1)	84 (13.0)	0.047
CKD stages ≥ 3, number (%)	27 (3.5)	1 (4.8)	1 (2.3)	3 (5.4)	22 (3.4)	0.84
Malignancy history, number (%)	39 (5.1)	3 (14.3)	2 (4.7)	3 (5.4)	31 (4.8)	0.28
CVD history, number (%)	72 (9.4)	2 (9.5)	3 (7.0)	12 (21.4)	55 (8.5)	0.020
Entry year 2001–2011 ^§^, number (%)	361 (47.1)	11 (52.4)	35 (81.4)	19 (33.9)	296 (45.8)	<0.001

Data were obtained when patients were diagnosed with bronchiectasis plus ILD, bronchiectasis alone, or ILD alone based on HRCT findings. For RA patients without bronchiectasis or ILD, data were obtained at the time of RA diagnosis. Patients with ILD and traction bronchiectasis on HRCT scans were not classified as having bronchiectasis plus ILD, but were classified as having ILD alone. * Differences among the patient groups were assessed using Fisher’s exact probability test with post hoc Holm test for categorical variables and one-way ANOVA with Tukey’s HSD post hoc test for continuous variables. For disease duration, we used the Kruskal–Wallis test with Tukey’s HSD post hoc test. Comparisons with *p* < 0.05 in post hoc tests are shown below. Age: *p* < 0.001 for BE+ILD vs. without BE or ILD, BE alone vs. without BE or ILD, ILD alone vs. without BE or ILD. Male: *p* < 0.001 for ILD alone vs. BE alone and ILD alone vs. without BE or ILD; *p* = 0.001 for BE+ILD vs. without BE or ILD; *p* = 0.003 for BE+ILD vs. BE alone. Disease duration: *p* < 0.001 for BE+ILD vs. ILD alone and BE alone vs. without BE or ILD; *p* = 0.014 for BE+ILD vs. without BE or ILD. Steinbrocker stage III/IV: *p* < 0.001 for BE alone vs. without BE or ILD; *p* = 0.001 for BE alone vs. ILD alone; *p* = 0.030 for BE alone vs. BE+ILD. Anti-CCP positive: *p* = 0.023 for ILD alone vs. without BE or ILD. RF positive: *p* = 0.004 for ILD alone vs. without BE or ILD; *p* = 0.021 for BE alone vs. without BE or ILD. Smoking ≥ 30 pack-years: *p* < 0.001 for BE+ILD vs. without BE or ILD, ILD alone vs. BE alone, and ILD alone vs. without BE or ILD; *p* = 0.014 for BE+ILD vs. BE alone. BMI < 18.5: *p* < 0.001 for BE+ILD vs. without BE or ILD and BE alone vs. without BE or ILD; *p* = 0.045 for ILD alone vs. without BE or ILD. Type 2 diabetes: *p* = 0.040 for BE alone vs. ILD alone. CVD history: *p* = 0.004 for ILD alone vs. without BE or ILD. Entry prior to 2011: *p* < 0.001 for BE alone vs. ILD alone and BE alone vs. without ILD or BE; *p* = 0.021 for BE alone vs. BE+ILD. ^†^ Defined as the time length from onset of articular symptoms and/or signs, which was obtained from patient self-reports. ^‡^ Patients who had a smoking history stopped smoking based on smoking cessation instructions following RA diagnosis. ^§^ During this period, biological DMARDs were available, but JAK inhibitors were not available in our institution. ^¶^ Sixty-eight patients were classified as UIP (18 in the bronchiectasis plus ILD group and 50 in the ILD alone group) and 9 patients were diagnosed as NSIP (3 in the bronchiectasis plus ILD group and 6 in the ILD alone group). Abbreviations: ANOVA, analysis of variance; anti-CCP, anti-cyclic citrullinated peptide antibodies; BE, bronchiectasis; BMI, body mass index; CKD, chronic kidney disease; CVD cardiovascular disease; DAS28-CRP, 28-joint disease activity score using C-reactive protein; DMARDs, disease-modifying antirheumatic drugs; HRCT, high-resolution computed tomography; HSD, honestly significant difference; ILD, interstitial lung disease; JAK, Janus kinase; RA, rheumatoid arthritis; RF, rheumatoid factor; SD, standard deviation.

**Table 2:** Characteristics of HRCT-proven bronchiectasis in RA patients.

	Total	Bronchiectasis Plus ILD	Bronchiectasis Alone	
Characteristic	*(n* = 64)	(*n* = 21)	(*n* = 43)	*p* *
Onset of bronchiectasis, patient number (%)				
Simultaneous diagnosis with RA	19 (29.7)	8 (38.1)	11 (25.6)	0.39
Within 3 years after RA diagnosis	17 (26.6)	5 (23.8)	12 (27.9)	1.00
>3 years and ≤5 years after RA diagnosis	10 (15.6)	1 (4.8)	9 (20.9)	0.15
>5 years after RA diagnosis	18 (28.1)	7 (33.3)	11 (25.6)	0.56
Bronchiectasis severity index (BSI, range 0–26), mean (SD)	5.0 (1.9)	5.1 (1.8)	4.5 (2.1)	0.30
Mild (≤4 points), patient number (%)	23 (35.9)	4 (19.0)	19 (44.2)	0.058
Moderate (5–8 points), patient number (%)	40 (62.5)	17 (81.0)	23 (53.4)	0.053
Severe (≥9 points), patient number (%)	1 (1.6)	0	1 (2.3)	0.55
Clinically significant bronchiectasis, patient number (%)	17 (26.6)	4 (19.0)	13 (30.2)	0.39
MRC dyspnea score, mean (SD)	0.09 (0.43)	0 (0)	0.14 (0.52)	0.22
*Pseudomonas* colonization ^†^, patient number (%)	1 (1.6)	0	1 (2.3)	1.00
HRCT scans ^‡^				
Bronchiectasis				
Number of lobes involved ^§^, mean (SD)	5.2 (1.4)	5.3 (1.4)	5.2 (1.3)	0.91
Cystic bronchiectasis, patient number (%)	0	0	0	–
Centrilobular nodules				
Number of patients (%)	48 (75)	10 (47.6)	38 (88.3)	<0.001
Number of lobes involved ^§^, mean (SD)	3.7 (1.7)	2.8 (1.7)	4.0 (1.7)	0.059
Mosaic attenuation				
Number of patients (%)	8 (12.5)	6 (28.6)	2 (4.7)	0.012
Number of lobes involved ^§^, mean (SD)	4.3 (2.0)	4.3 (2.0)	4.0 (2.8)	0.86
Bronchial wall thickness, patient number (%)				
Number of patients (%)	61 (95.3)	20 (95.2)	41 (95.3)	1.00
Severity (0–4), mean (SD)	1.6 (0.6)	1.5 (0.6)	1.6 (0.7)	0.73
Emphysema ^¶^				
Number of patients (%)	9 (14.1)	6 (28.6)	3 (7.0)	0.049
Pulmonary function test ^††^, mean (SD)				
FVC, % predicted	90.4 (20.1)	93.3 (16.5)	89.1 (21.7)	0.45
FEV_1_, % predicted	101.5 (22.5)	115.1 (19.4)	94.8 (21.1)	<0.001
FEV_1_/FVC ratio (%)	78.4 (8.8)	79.3 (8.0)	76.8 (11.4)	0.50
MMF, % predicted	60.1 (31.0)	78.1 (32.8)	51.4 (26.2)	0.001
DL_CO_, % predicted	89.5 (25.9)	78.9 (21.1)	95.2 (26.8)	0.025

Data were obtained when patients were diagnosed with bronchiectasis plus ILD or bronchiectasis alone. Patients with ILD and traction bronchiectasis on HRCT scans were not classified as having bronchiectasis plus ILD. * Differences among the patient groups were assessed using Fisher’s exact probability test for categorical variables and independent-measures *t*-tests for continuous variables. For MRC score, the Mann–Whitney U test was used. ^†^ Other colonizing bacteria were not identified at the diagnosis of bronchiectasis. ^‡^ The k coefficients were 0.636 for bronchiectasis, 0.712 for centrilobular nodules, 0.717 for mosaic attenuation, 0.918 for bronchiolar wall thickness, and 1.000 for emphysema, indicating substantial agreement in radiographic scoring between readers. ^§^ Lingula is considered to be a separate lobe. ^¶^ Emphysema was present in 53.6% of patients with ILD alone (30 of 56 patients). ^††^ Expressed as a ratio of measured to predicted values (% predicted) except for FEV_1_/FVC ratio. Abbreviations: DL_CO_, diffusing capacity of carbon monoxide; FEV_1_, forced expiratory volume in one second; FVC, forced vital capacity; HRCT, high-resolution computed tomography; ILD, interstitial lung disease; MMF, maximal mid-expiratory flow; MRC, Medical Research Council; RA, rheumatoid arthritis; SD, standard deviation.

**Table 3:** Mortality in RA patients, grouped by HRCT-based diagnosis of lung disease.

	Total	Bronchiectasis Plus ILD	Bronchiectasis Alone	ILD Alone	Without ILD or Bronchiectasis
Variable	(*n* = 766)	*(n* = 21)	(*n* = 43)	(*n* = 56)	*(n* = 646)
Follow-up ^†^, years, mean (SD)	9.7 (5.2)	6.4 (4.2)	7.7 (4.6)	6.5 (4.5)	10.2 (5.2)
Follow-up ^†^, years, median (IQR)	9.3 (5.7–13.7)	6.7 (2.1–10.7)	7.3 (4.3–11.5)	6.2 (2.4–8.9)	9.9 (6.1–14.0)
Lost to follow-up, number (%)	128 (16.7)	0	3 (7.0)	3 (5.4)	122 (18.9)
Death, number (%)	144 (18.8)	15 (71.4)	23 (53.5)	25 (44.6)	81 (12.5)
Crude incidence rate of death per 100 PYs (95% CI)	1.9 (1.6–2.3)	11.1 (6.7–18.4)	7.0 (4.6–10.5)	6.8 (4.6–10.1)	1.2 (1.0–1.5)

^†^ Follow-up began when patients were diagnosed with bronchiectasis plus ILD, bronchiectasis alone, or ILD alone. For RA patients without bronchiectasis or ILD, follow-up began with the diagnosis of RA. Abbreviations: 95% CI, 95% confidence interval; HRCT, high-resolution computed tomography; ILD, interstitial lung disease; IQR, interquartile range; PYs, person-years; RA, rheumatoid arthritis; SD, standard deviation.

**Table 4:** SMRs in RA patients grouped according to HRCT-based diagnosis of lung disease.

	Number of Deaths		SMR (95% CI) *
Patient Group	Observed	Expected	
All patients (*n* = 766)	144	111.8	1.29 (1.08–1.50)
Patients with bronchiectasis plus ILD (*n* = 21)	15	8.1	1.86 (0.92–2.80)
Bronchiectasis alone (*n* = 43)	23	6.7	3.43 (2.03–4.83)
ILD alone (*n* = 56)	25	10.3	2.42 (1.47–3.37)
Patients without bronchiectasis or ILD (*n* = 646)	81	86.7	0.93 (0.73–1.14)
Patients with bronchiectasis (*n* = 64) ^†^	38	14.8	2.57 (1.76–3.39)
Patients with ILD (*n* = 77) ^‡^	40	18.4	2.17 (1.50–2.85)

* Compared with the general population of Japan from 2001 to 2023. ^†^ Included patients with bronchiectasis plus ILD and patients with bronchiectasis alone. ^‡^ Included patients with bronchiectasis plus ILD and patients with ILD alone. Abbreviations: HRCT, high-resolution computed tomography; ILD, interstitial lung disease; RA, rheumatoid arthritis; SMR, standardized mortality ratio.

**Table 5:** Cause of death in RA patients, grouped according to the HRCT-based diagnosis of lung disease.

	Total	Bronchiectasis Plus ILD	Bronchiectasis Alone	ILD Alone	Without ILD or Bronchiectasis	
Cause of Death	(*n* = 144)	(*n* = 15)	(*n* = 23)	(*n* = 25)	(*n* = 81)	*p* *
Age at death, years (SD)	78.5 (9.5)	83.5 (6.9)	78.7 (8.2)	79.7 (8.8)	77.1 (10.3)	0.10
Malignancy, number (%)	46 (31.9)	7 (46.7)	5 (21.7)	6 (24.0)	28 (34.6)	0.31
Lung cancer, number (%)	13 (9.0)	6 (40.0)	0	4 (16.0)	3 (3.7)	<0.001
Respiratory failure, number (%)	15 (10.4)	4 (26.7)	3 (13.0)	8 (32.0)	0	<0.001
Exacerbation of ILD ^†^, number (%)	12 (8.3)	4 (26.7)	0	8 (32.0)	0	<0.001
Exacerbation of BE, number (%)	3 (2.1)	0	3 (13.0)	0	0	0.001
Serious infection, number (%)	18 (12.5)	1 (6.7)	4 (17.4)	4 (16.0)	9 (11.1)	0.71
Lung infection ^‡^, number (%)	13 (9.0)	0	4 (17.4)	4 (16.0)	5 (6.2)	0.13
Cardiovascular events, number (%)	27 (18.8)	0	7 (30.4)	2 (8.0)	18 (22.2)	0.045
Death of old age, number (%)	17 (11.8)	2 (13.3)	3 (13.0)	3 (12.0)	9 (11.1)	0.99
Aspiration pneumonia, number (%)	7 (4.9)	1 (6.7)	1 (4.3)	1 (4.0)	4 (4.9)	0.98
Others, number (%)	14 (9.7)	0	0	1 (4.0)	13 (16.0)	0.033

* Differences among the patient groups were assessed using Fisher’s exact probability test with post hoc Holm test for categorical variables and one-way ANOVA with Tukey’s HSD post hoc test for continuous variables. Comparisons with *p* < 0.05 in post hoc tests are shown below: Lung cancer: *p* < 0.001 for BE+ILD vs. without BE or ILD; *p* = 0.002 for BE+ILD vs. BE alone. Respiratory failure: *p* < 0.001 for ILD alone vs. without BE or ILD; *p* = 0.002 for BE alone vs. without BE or ILD; *p* = 0.003 for BE+ILD vs. without BE or ILD. Exacerbation of ILD: *p* < 0.001 for ILD vs. without BE or ILD; *p* = 0.003 for BE+ILD vs. without BE or ILD; *p* = 0.004 for BE vs. ILD; *p* = 0.018 for BE+ILD vs. BE. Exacerbation of BE: *p* = 0.002 for BE vs. without BE+ILD. Cardiovascular events: *p* = 0.028 for BE+ILD vs. BE alone. Others: *p* = 0.004 for BE vs. without BE or ILD. ^†^ Eleven patients were diagnosed as UIP (3 in the bronchiectasis plus ILD group and 8 in the ILD alone group). ^‡^ *Pneumocystis* pneumonia was the cause of death in 1 patient with BE alone, 3 patients with ILD alone, and 1 patient without BE or ILD. Bacterial infection was the cause of death in 3 patients with BE alone, 1 patient with ILD alone, and 4 patients without BE or ILD. Abbreviations: ANOVA, analysis of variance; BE, bronchiectasis; HSD, honestly significant difference; HRCT, high-resolution computed tomography; ILD, interstitial lung disease; NTM, nontuberculous mycobacteria; RA, rheumatoid arthritis; SD, standard deviation.

**Table 6:** Predictive factors for death in overall RA patients.

	Univariable Analysis		Multivariable Analysis	
Variables at Baseline	Unadjusted HR (95% CI)	*p* *	Adjusted HR (95% CI)	*p* *
Age per additional year	1.11 (1.09–1.13)	<0.001	1.10 (1.08–1.12)	<0.001
Male vs. female	2.06 (1.47–2.89)	<0.001	1.43 (0.92–2.24)	0.12
Disease duration per additional year	1.02 (1.01–1.04)	0.008	0.99 (0.97–1.00)	0.10
DAS28-CRP per additional unit	0.83 (0.74–0.93)	0.002	1.11 (0.98–1.26)	0.090
Steinbrocker stage III/IV vs. I/II	1.28 (0.92–1.77)	0.15	–	–
Anti-CCP, positive vs. negative	2.27 (1.11–4.64)	0.024	2.44 (0.80–7.50)	0.12
RF, positive vs. negative	2.10 (1.03–4.28)	0.042	1.05 (0.34–3.23)	0.94
HRCT-based diagnosis of lung disease				
Without bronchiectasis or ILD	1 (reference)	–	1 (reference)	–
ILD alone	6.60 (4.19–10.41)	<0.001	3.60 (2.19–5.93)	<0.001
Bronchiectasis alone	6.45 (4.03–10.33)	<0.001	4.43 (2.56–7.66)	<0.001
Bronchiectasis plus ILD	11.08 (6.31–19.43)	<0.001	4.12 (2.13–7.95)	<0.001
Smoking ≥ 30 pack-years vs. no history	2.33 (1.59–3.42)	<0.001	1.15 (0.69–1.89)	0.60
BMI < 18.5, yes vs. no	2.95 (2.00–4.35)	<0.001	2.84 (1.86–4.34)	<0.001
Entry year 2001–2011, yes vs. no	1.49 (1.02–2.19)	0.042	1.38 (0.92–2.08)	0.12

* Univariable and multivariable Cox proportional hazards regression analyses were conducted to evaluate the effect of patient characteristics on death outcome in overall RA patients. For RA patients with bronchiectasis and/or ILD, the index date was defined as the time of diagnosis of each pulmonary disease. For RA patients without either pulmonary complication, the index date was defined as the time of diagnosis of RA. As baseline variables, we used patient characteristics obtained at the index date defined for each patient group. All variables with *p*-values < 0.10 in the univariable models were introduced into multivariable analysis using a forced entry procedure. Abbreviations: anti-CCP, anti-cyclic citrullinated peptide antibodies; BMI, body mass index; 95% CI, 95% confidence interval; DAS28-CRP, 28-joint disease activity score using C-reactive protein; HR, hazard ratio; HRCT, high-resolution computed tomography; ILD, interstitial lung disease; RA, rheumatoid arthritis; RF, rheumatoid factor.

**Table 7:** Predictive factors for death in overall RA patients (sensitivity analysis).

	Univariable Analysis		Multivariable Analysis	
Variables at Baseline	Unadjusted HR (95% CI)	*p* *	Adjusted HR (95% CI)	*p* *
Age per additional year	1.11 (1.09–1.13)	<0.001	1.10 (1.08–1.12)	<0.001
Male vs. female	2.10 (1.49–2.95)	<0.001	1.51 (0.97–2.36)	0.070
Disease duration per additional year	1.01 (0.99–1.03)	0.25	–	–
DAS28-CRP per additional unit	1.00 (0.88–1.13)	0.98	–	–
Steinbrocker stage III/IV vs. I/II	1.15 (0.82–1.60)	0.42	–	–
Anti-CCP, positive vs. negative	2.13 (1.05–4.36)	0.037	2.60 (0.89–7.56)	0.080
RF, positive vs. negative	1.92 (0.94–3.93)	0.073	0.97 (0.33–2.83)	0.95
HRCT-based diagnosis of lung disease				
Without bronchiectasis or ILD	1 (reference)	–	1 (reference)	–
ILD alone	5.80 (3.69–9.12)	<0.001	3.16 (1.92–5.22)	<0.001
Bronchiectasis alone	3.50 (2.20–5.56)	<0.001	2.37 (1.48–3.82)	<0.001
Bronchiectasis plus ILD	5.47 (3.09–9.67)	<0.001	1.62 (0.89–2.94)	0.12
Smoking ≥ 30 pack-years vs. no history	2.30 (1.57–3.39)	<0.001	1.16 (0.71–1.92)	0.55
BMI < 18.5, yes vs. no	2.74 (1.86–4.05)	<0.001	2.68 (1.75–4.11)	<0.001
Entry year 2001–2011, yes vs. no	1.25 (0.84–1.84)	0.27	–	–

* We performed univariable and multivariable Cox proportional hazards regression analyses of the overall RA patient population, using the common index date (time of RA diagnosis) and treating the diagnosis of each pulmonary disease as a time-varying exposure variable. Baseline variables were obtained at the index date (time of RA diagnosis) for all patient groups. All variables with *p*-values < 0.10 in the univariable models were introduced into multivariable analysis using a forced entry procedure. Abbreviations: anti-CCP, anti-cyclic citrullinated peptide antibodies; BMI, body mass index; 95% CI, 95% confidence interval; HR, hazard ratio; HRCT, high-resolution computed tomography; ILD, interstitial lung disease; RA, rheumatoid arthritis; RF, rheumatoid factor.

**Table 8:** Predictive factors for death in RA patients with bronchiectasis.

	Univariable Analysis		Multivariable Analysis	
Variables at Baseline	Unadjusted HR (95% CI)	*p* *	Adjusted HR (95% CI)	*p* *
Age per additional year ^†^	1.07 (1.03–1.12)	<0.001 ^†^	–	–
Male vs. female	1.78 (0.92–3.44)	0.089	1.40 (0.67–2.95)	0.37
Disease duration per additional year	0.98 (0.94–1.02)	0.32	–	–
DAS28-CRP per additional unit	0.87 (0.67–1.12)	0.28	–	–
Steinbrocker stage III/IV vs. I/II	0.64 (0.32–1.25)	0.19	–	–
Anti-CCP, positive vs. negative	0.42 (0.13–1.40)	0.16	–	–
RF, positive vs. negative	0.44 (0.10–1.85)	0.26	–	–
Smoking ≥ 30 pack-years vs. no history	1.74 (0.81–3.70)	0.15	–	–
BMI < 18.5, yes vs. no ^†^	2.29 (1.22–4.29)	<0.001 ^†^	–	–
Entry year 2001–2011, yes vs. no	1.48 (0.64–3.41)	0.36	–	–
Simultaneous diagnosis, yes vs. no	0.65 (0.29–1.41)	0.27	–	–
BSI per additional unit	1.38 (1.21–1.56)	<0.001	1.37 (1.20–1.58)	<0.001
Clinically significant bronchiectasis, yes vs. no	1.39 (0.72–2.68)	0.32	–	–
MRC dyspnea score per additional unit ^†^	1.67 (0.92–3.03)	0.093 ^†^	–	–
Presence of ILD, yes vs. no	1.79 (0.93–3.48)	0.084	1.05 (0.49–2.23)	0.89
Bronchiectasis ≥3 lobes involved, yes vs. no	1.55 (0.48–5.03)	0.47	–	–
FEV_1_ > 80%, yes vs. no	0.96 (0.44–2.09)	0.91	–	–

* Univariable and multivariable Cox proportional hazards regression analyses were conducted for RA patients with HRCT-proven bronchiectasis. The index date was defined as the time of diagnosis of bronchiectasis because RA and ILD were diagnosed before or simultaneously with the diagnosis of bronchiectasis. Baseline variables were obtained at the index date (time of diagnosis of bronchiectasis). Variables with *p*-values < 0.10 in the univariable models were introduced into multivariable analysis as predictor variables using a forced entry procedure. ^†^ We introduced BSI into multivariable regression analysis as one of the predictor variables with *p*-values < 0.10. Age, BMI, or MRC dyspnea score could not be used together with BSI, because these variables were the components of BSI. Abbreviations: anti-CCP, anti-cyclic citrullinated peptide antibodies; BMI, body mass index; BSI, bronchiectasis severity index; 95% CI, 95% confidence interval; DAS28-CRP, 28-joint disease activity score using C-reactive protein; FEV_1_, forced expiratory volume in 1 s; HR, hazard ratio; ILD, interstitial lung disease; MRC, Medical Research Council; RA, rheumatoid arthritis; RF, rheumatoid factor.

## Data Availability

All data supporting the findings are available from the Human Research Ethics Committee of National Hospital Organization Kumamoto Saishun Medical Center for all interested researchers who meet the criteria for access to confidential data. Because these data include patients’ personal information, the Committee does not recommend that such data be made public unnecessarily. Please contact Mr. Masahiro Hamaguchi, Control Manager of the Committee, at 616-syol@mail.hosp.go.jp to request the data.

## Supplementary Materials

The following supporting information can be downloaded at https://www.mdpi.com/article/10.3390/jcm15218703/s1. Table S1. Bronchiectasis severity index of RA patients with HRCT-proven bronchiectasis. Table S2. DMARD use during follow-up after the diagnosis of pulmonary conditions.
