# Supplementary material for: Impact of Bronchiectasis on Long-Term Mortality in Patients with Rheumatoid Arthritis: A Real-World Retrospective Cohort Study
Source: J Clin Med. 2026 Nov 7;15(21):8703. doi: 10.3390/jcm15218703 (PMC13763738; doi:10.3390/jcm15218703)
Supplement: Supplementary file 1 [file jcm-15-08703-s001.zip › jcm-4633074-supplementary.pdf]

Table S1. Bronchiectasis severity index of RA patients with HRCT-proven bronchiectasis

Severity marker	Score	Bronchiectasis plus ILD (n = 21)	Bronchiectasis alone (n = 43)	p *
Age, years				
<50	0	1 (4.8)	1 (2.3)	1.00
50–69	2	3 (14.3)	20 (46.5)	0.014
70–79	4	13 (61.9)	15 (34.9)	0.060
≥80	6	4 (19.0)	7 (16.3)	1.00
BMI, kg/m^2				
<18.5	2	7 (33.3)	13 (30.2)	1.00
FEV_1, % predicted		115.1 (19.4)	94.8 (21.1)	<0.001
>80%	0	19 (90.5)	34 (79.1)	0.31
50–80%	1	2 (9.6)	8 (18.6)	0.48
30–49%	2	0	1 (2.3)	1.00
<30%	3	0	0	–
Severe exacerbation in previous year †				
Yes	5	0	0	–
Exacerbation frequency in previous year				
0	0	21 (100)	43 (100)	1.00
1–2	0	0	0	–
3 or more	2	0	0	–
MRC dyspnea score				
0–3	0	21 (100)	40 (93.0)	0.55
4	2	0	3 (7.0)	0.55
5	3	0	0	–
Pseudomonas aeruginosa colonization				
Yes	3	0	1 (2.3)	1.00
Colonization with other microorganisms				
Yes	1	0	0	–
Radiological severity (≥3 lobes involved or cystic changes)				
Yes	1	19 (90.5)	38 (88.4)	1.00
Bronchiectasis severity index, mean (SD)	–	5.1 (1.8)	4.5 (2.1)	0.30

Data were obtained when patients were diagnosed with bronchiectasis plus ILD or bronchiectasis alone. Data are expressed as number (%) of patients.

* Differences among the patient groups were assessed using Fisher's exact probability test.

† Severe exacerbation was defined as requiring hospitalization or intravenous antibiotic treatment.

Abbreviations: BMI, body mass index; FEV_1, forced expiratory volume in 1 second; HRCT, high-resolution computed tomography; ILD, interstitial lung disease; MRC, Medical Research Council; RA, rheumatoid arthritis.

Table S2. DMARD use during follow-up after the diagnosis of pulmonary conditions.

Variable	Total (*n* = 766)	Bronchiectasis plus ILD (*n* = 21)	Bronchiectasis alone (*n* = 43)	ILD alone (*n* = 56)	Without ILD or bronchiectasis (*n* = 646)	*p* *
DMARD use, number (%)						
MTX	658 (85.9)	8 (38.1)	33 (76.7)	34 (60.7)	583 (90.2)	<0.001
Other csDMARDs †	252 (32.9)	13 (61.9)	18 (41.9)	29 (51.8)	192 (29.8)	<0.001
bDMARDs						
TNF inhibitors	370 (48.3)	8 (38.1)	17 (39.5)	29 (51.8)	316 (49.0)	0.46
IL-6 inhibitors	259 (33.8)	8 (38.1)	16 (37.2)	32 (55.4)	203 (31.4)	0.001
Abatacept	130 (17.0)	6 (28.6)	14 (32.6)	20 (35.7)	90 (13.9)	<0.001
JAK inhibitors ‡	284 (37.1)	6 (28.6)	9 (20.9)	21 (37.5)	248 (38.4)	0.12
Length of exposure, years, mean (SD)						
MTX	8.1 (6.8)	5.2 (3.7)	6.1 (4.5)	5.5 (4.9)	8.4 (7.0)	0.018
Other csDMARDs †	4.5 (3.8)	4.3 (5.2)	5.1 (4.1)	3.9 (3.2)	4.6 (3.8)	0.76
bDMARDs						
TNF inhibitors	3.9 (4.4)	2.7 (3.0)	4.0 (4.0)	3.4 (3.8)	4.0 (4.4)	0.77
IL-6 inhibitors	3.1 (3.4)	2.4 (2.9)	2.2 (2.5)	2.2 (3.2)	3.4 (3.5)	0.15
Abatacept	2.7 (3.0)	3.8 (3.7)	3.2 (3.3)	1.6 (1.8)	2.8 (3.0)	0.25
JAK inhibitors ‡	3.4 (2.9)	2.3 (2.9)	4.2 (2.3)	2.6 (2.5)	3.5 (3.0)	0.33

* Differences among the patient groups were assessed using Fisher's exact probability test with *post hoc* Holm test for categorical variables and one-way ANOVA with Tukey's HSD *post hoc* test for continuous variables. Comparisons with $p < 0.05$ in *post hoc* tests are shown below.

MTX use: $p < 0.001$ for BE+ILD vs. without BE or ILD and ILD alone vs. without BE or ILD; $p = 0.005$ for BE+ILD vs. BE alone; $p = 0.017$ for BE alone vs. without BE or ILD.

Other csDMARDs: $p = 0.001$ for ILD alone vs. without BE or ILD; $p = 0.003$ for BE+ILD vs. without BE or ILD.

IL-6 inhibitor: $p < 0.001$ for ILD alone vs. without BE or ILD.

Abatacept: $p < 0.001$ for ILD alone vs. without BE or ILD; $p = 0.003$ BE alone vs. without BE or ILD.

† Included tacrolimus, leflunomide, sulfasalazine, and bucillamine.

‡ Included tofacitinib, peficitinib, baricitinib, upadacitinib, and filgotinib.

Abbreviations: ANOVA, analysis of variance; bDMARDs, biological DMARDs; BE, bronchiectasis; csDMARDs, conventional synthetic DMARDs; DMARDs, disease-modifying antirheumatic drugs; HSD, honestly significant difference; IL-6, interleukin-6; ILD, interstitial lung disease; JAK, Janus kinase; MTX, methotrexate; RA, rheumatoid arthritis; SD, standard deviation; TNF, tumor necrosis factor.
